# Limitations of procedural learning curves: reassessment of the clinical relevance of learning curve analysis in robotic right hemicolectomy

**DOI:** 10.1007/s11701-026-03970-w

**Published:** 2026-09-24

**Authors:** Alexander Wilk, Anna-Marie Wilk, Hatice Altin, Thorsten Brechmann, Metin Mazgaldzhi, Benno Mann

**Affiliations:** 1 Faculty of Medicine, University of Tübingen, Tübingen, Germany; 2 Department of General, Visceral and Transplant Surgery, University Hospital Tübingen, Hoppe-Seyler-Straße 3, 72076 Tübingen, Germany; 3 Wroclaw Medical University, Wroclaw, Poland; 4 Ruhr University Bochum, Bochum, Germany; 5 Department of Gastroenterology and Oncology, Knappschaft Kliniken Bottrop, Bottrop, Germany; 6 Department of General, Visceral and Robotic Surgery, Augusta-Klinikum Bochum Mitte, Bochum, Germany

**Keywords:** Minimally invasive surgery, Colorectal surgery, Oncology, Learning curves

## Abstract

Learning curves in robotic right hemicolectomy (RRH) are assessed using cumulative sum (CUSUM) analysis. CUSUM was originally designed for industrial processes using external benchmarks. Medical applications rely on internal benchmarks, typically the cohort-specific median. This introduces circularity, obscures early deviations and distorts inflection points. In addition, phase definitions are based on procedural metrics with limited linkage to clinical outcomes. Current models may not reflect surgical proficiency. Data were drawn from a prospectively maintained institutional database covering RRH procedures performed between 2010 and 2025; after exclusions, 229 cases remained for analysis. Cases were split into a training group (TG; *n* = 164) and a control group (CG; *n* = 65), the latter comprising procedures by two surgeons already in their mastery phase and used as an external benchmark (EB). Learning-curve analysis of RRH referenced against this EB formed the primary endpoint, while RA-CUSUM analysis and perioperative outcome parameters were assessed as secondary endpoints. Application of the external benchmark approach revealed a prolonged initial learning phase (approximate transition zone 35–55 cases). Major complications, conversion rate, blood loss, and length of stay showed the steepest changes initially, with stabilization thereafter, while lymph node yield reached an early plateau. Operative time demonstrated a continuous increase throughout. Compared with previously reported analyses, learning phases were extended and showed different patterns when clinical outcomes were considered. The prolonged first learning phase for RRH indicates delayed perioperative proficiency. Learning curve analysis should shift to a clinically driven approach with external benchmark methodology. Benchmark values are institution-specific; the methodology may serve as a conceptual framework for future analyses.

**Graphical abstract:**

**Figure:**
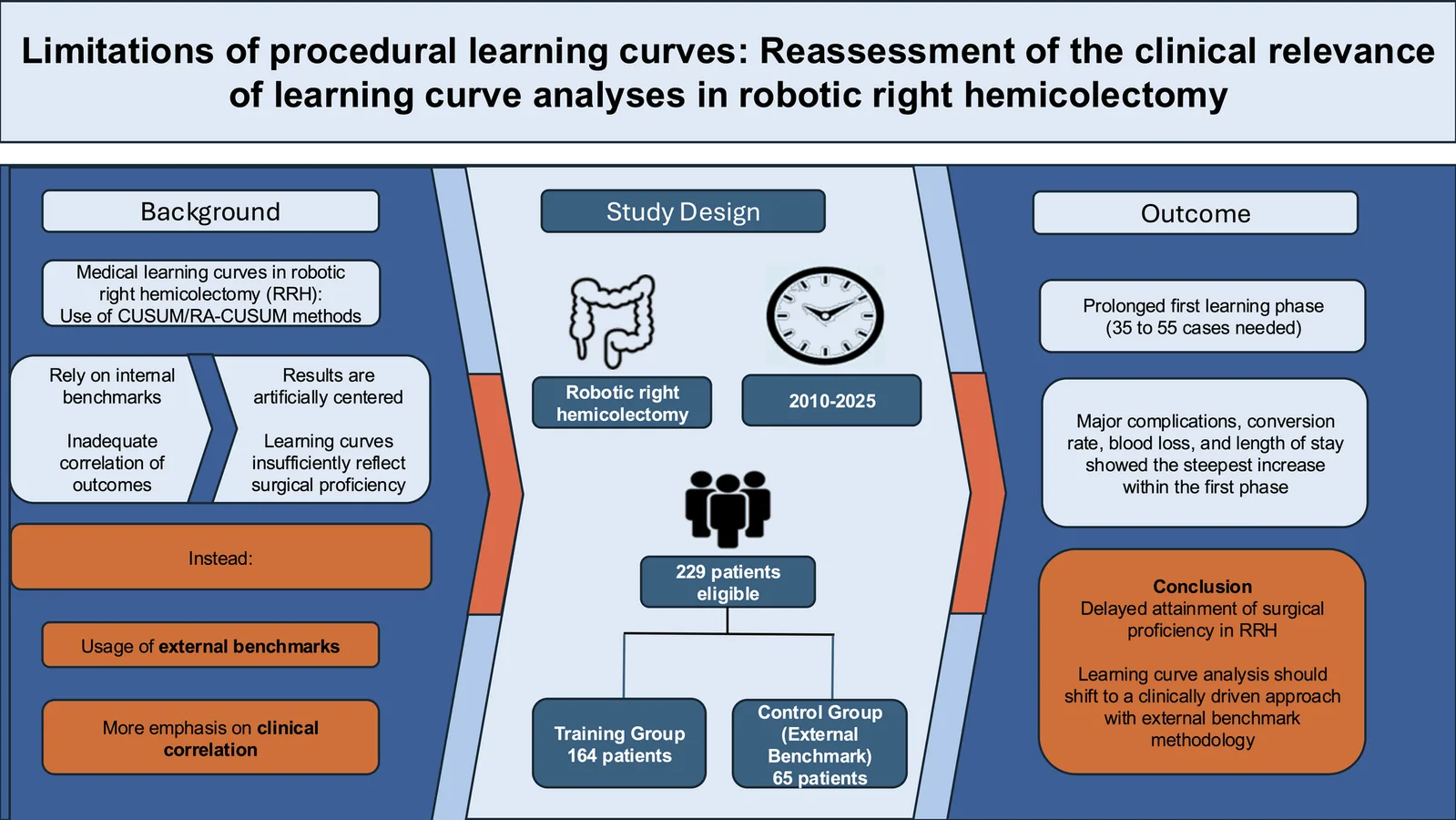

**Supplementary Information:**

The online version contains supplementary material available at https://doi.org/10.1007/s11701-026-03970-w.

## Introduction

Surgical resection forms the cornerstone of curative treatment for right-sided colon cancer. Oncologic robotic right hemicolectomy (RRH) represents a relatively novel and technically complex procedure within colorectal surgery, owing to demanding anatomical planes, central vascular ligation and the requirement for oncologic approach [1–7]. To achieve procedural proficiency, several learning curves for RRH have been described [2, 8–15]. A widely accepted means of the surgeon’s progress is described [16] by the cumulative sum (CUSUM) methodology [2, 17–20]. CUSUM was originally developed for industrial process control, where analyses are conducted against externally defined benchmarks [15, 21]. Such benchmarks represent predefined reference values independent of the dataset under investigation. In contrast, medical CUSUM analyses typically rely on an internal benchmark, most commonly the cohort-specific median, as the reference value [19, 22]. Phase classification is frequently based on case numbers, with emphasis on surrogate metrics such as operative time or blood loss, often without adequate linkage to patient-centered outcomes [20, 23].

This methodological approach raises two principal concerns. Firstly, the internal benchmark for RRH is derived from the same dataset that is subsequently analyzed, early and late cases contribute equally to its calculation [17–19]. As a consequence, CUSUM curves become artificially centered, early deviations may be dampened and potential inflection points may be distorted. Hence, a larger data set shifts the median to later cases [19]. From a methodological standpoint, the use of an external benchmark from independent reference cohorts is therefore statistically more robust. Secondly, a key unresolved issue is the identification of a clinically meaningful learning curve phase. In routine practice, medical CUSUM analysis for RRH typically reflects a purely process based phase classification. While this approach reflects operative efficiency, it does not permit reliable conclusions in the absence of outcome- and morbidity-oriented endpoints required to substantiate clinical improvement [2, 24, 25]. Consequently, learning curve phase classification should evolve from a purely process-based construct toward a more outcome oriented framework.

In the light of these methodological limitations, current phase definitions for RRH may underestimate true surgical proficiency. Therefore, the aim of the present study was to analyze the learning curves of robotic right hemicolectomy using a prospectively maintained institutional database with an externally derived benchmarking approach. Furthermore, increased emphasis is given to the association between learning curves and clinical outcomes such as major complication and length of stay.

## Methods

### Patients and study design

**Fig. 1:**
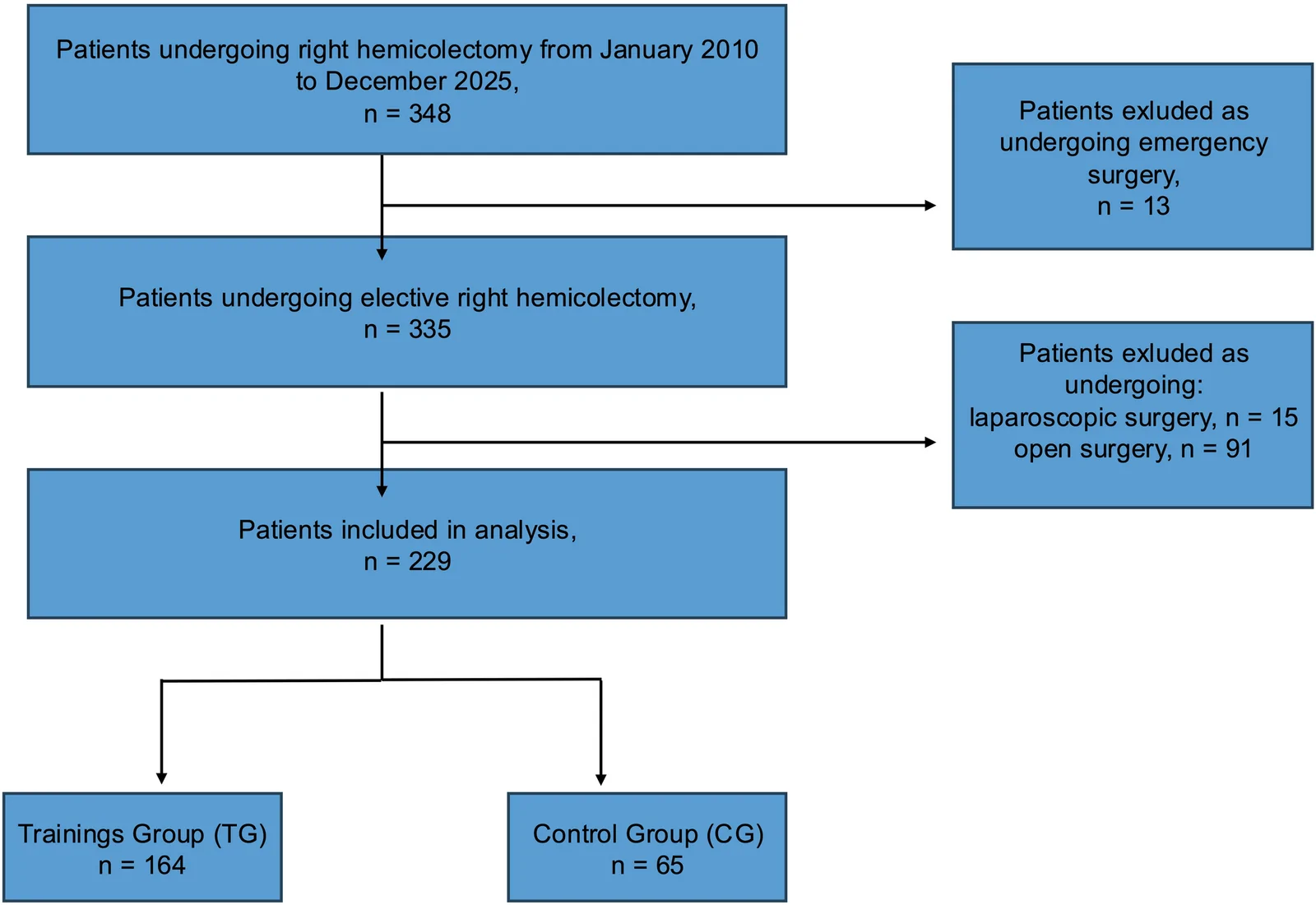
Flow chart of patient inclusion

Data for this retrospective analysis were drawn from an institutional, prospectively maintained colorectal surgery registry at Augusta Academic Hospital Bochum, Germany. All patients undergoing robotic right hemicolectomy (RRH) for an oncologic indication between January 2010 and December 2025 were considered eligible. The choice of operative approach and the extent of resection followed multidisciplinary tumor-board discussion, taking into account tumor location, histopathological findings and each patient’s individual clinical condition. Among 348 patients identified in the registry, cases treated emergently or via an open or laparoscopic approach were excluded, yielding a final analytic cohort of 229 RRH patients (Fig. 1). A subset of this cohort was also included in an earlier report from our group comparing perioperative and oncologic outcomes of robotic versus open right hemicolectomy at this institution [26]; that publication addressed a distinct research question and did not examine learning curves or a benchmark-based analysis, so the present investigation should be regarded as complementary rather than overlapping in scope.

### Variables and data sources

Baseline demographic and clinical characteristics recorded for each patient included age, sex, body mass index (BMI), ECOG performance status, ASA class, history of prior abdominal surgery, prior chemotherapy, and preoperative laboratory results. Intraoperatively, we captured operative time (OR-time), estimated blood loss, adverse events graded per the ClassIntra system [27], any concomitant resections, conversion to open surgery, transfusion requirements, epidural catheter use, and stoma formation. Postoperative variables comprised ICU length of stay, total hospital stay, morbidity graded I–V on the Clavien–Dindo scale, need for reintervention, anastomotic leakage, postoperative laboratory findings, and 30-day mortality. From the histopathology reports we extracted tumor entity, UICC stage, resection margin status, tumor diameter, and lymph-node yield (harvested and tumor-positive nodes).

### Study endpoints

The primary endpoint was learning curve analysis of RRH applying the external benchmark approach with correlation to clinical outcomes. Secondary endpoints comprised risk-adjusted CUSUM (RA-CUSUM) analysis and assessment of perioperative outcome parameters.

### Statistical methods

To enable the learning-curve analysis, the cohort was divided into a training group (TG; *n* = 164) and a control group (CG; *n* = 65). The TG comprised procedures performed by five surgeons between 2010 and 2023 and was used to trace learning trajectories at cohort level, whereas the CG consisted of cases carried out in 2024–2025 by two surgeons who had already reached mastery phase; the latter therefore constituted the external benchmark (EB). The two surgeons providing the external benchmark are among the five surgeons contributing to the training group; their own early robotic right hemicolectomies are therefore included in the training group. No procedures were excluded from the analysis on the basis of teaching or supervision status. The benchmark values reported here are institution-specific and are not intended as generalizable reference values; it is the external-benchmark methodology, rather than the absolute values derived at our institution, that is proposed as a conceptual framework. CUSUM methodology was applied to evaluate TG learning curves against this benchmark and to relate them to clinical outcomes. Board certification in colorectal surgery was held by every operating surgeon. Data were analyzed using IBM SPSS Statistics, version 31.0.0.0. As the Kolmogorov–Smirnov test indicated departure from a normal distribution (*p* < .001), non-parametric methods were applied throughout, with Cohen’s d and Phi reported as effect-size measures for continuous and categorical variables, respectively [28, 29]. Learning trajectories were characterized using CUSUM and RA-CUSUM analysis, from which phase-specific summary curves were derived and referenced against the predefined external benchmark. Because the underlying outcome parameters were measured on different scales, each curve was rescaled relative to its own baseline value and expressed dimensionlessly, preserving the direction of deviation from benchmark. Accordingly, upward deviations indicate performance below the external benchmark, whereas downward deviations indicate performance exceeding it. This convention applies to both the CUSUM and the RA-CUSUM analyses. Risk adjustment was performed using a multivariable logistic regression model incorporating age, BMI, ASA score, ECOG performance status, cardiovascular comorbidity, prior abdominal surgery, and tumor size; for each patient, this model yielded an individually predicted probability of an adverse outcome, which was entered into the analysis as the expected event rate. Performance of the risk-adjustment model was assessed by the C-statistic with 95% confidence intervals and by the Hosmer–Lemeshow goodness-of-fit test, with calibration displayed graphically (Online resource 1, Table S6 and Figure S4). Phase boundaries in CUSUM analysis are conventionally derived by visual inspection of slope changes, which entails a degree of subjective interpretation. To limit this subjectivity, visual assessment was complemented by objective change-point analysis. The presence of a change point in each curve was tested using the Davies test [30]. The number of change points (none, one or two) was selected by the Bayesian information criterion [31], and change-point locations were estimated by segmented, piecewise linear regression [32], with 95% confidence intervals obtained by residual bootstrap (Online resource 1, Table S8). To assess case-mix drift across the training-group case sequence, cases were ordered chronologically and stratified at case 55; in addition, each variable was correlated with the chronological case number using Spearman rank correlation (Online resource 1, Table S7).

### Risk-adjustment model for RA-CUSUM analysis

For each patient i, the individual event probability (p_i_) was estimated using a logistic regression model:

$$\begin{aligned} \log it(p_{i} ){\text{ }} = {\text{ }} & \ln (p_{i} /{\text{ }}(1{\text{ }} - {\text{ }}p_{i} )){\text{ }} = \beta _{0} {\text{ }} + \beta _{1} Age_{i} + \beta _{2} BMI_{i} + \beta _{3} ASA_{i} \\ & + \beta _{4} ECOG_{i} + \beta _{5} PAS_{i} + \beta _{6} VD_{i} + \beta _{7} TS_{i} \\ \end{aligned} $$

Where p_i_ denotes the expected probability of the respective event for patient i, β₀ represents the intercept, and β₁–β₇ represent the regression coefficients for the included covariates.

BMI = body mass index; ASA = American Society of Anesthesiologists; ECOG = Eastern Cooperative Oncology Group; PAS = previous abdominal surgery; VD = vascular disease; TS = tumor size.

### Definitions of outcome parameters

OR-time denoted the elapsed time from the first skin incision to completion of skin closure. Intraoperative blood loss [27] was quantified as the volume of fluid collected in the suction canister at the end of the procedure. Conversion was defined as an unplanned switch to open laparotomy for any reason other than specimen retrieval.

The ClassIntra system was used to grade intraoperative adverse events, with grade ≥ 3 designated as major [27]; postoperative morbidity was likewise staged using the Clavien–Dindo classification, major complications being defined as grade ≥ III [33]. Resection margin status was assigned according to the TNM classification system [16].

### Surgical technique

All procedures were performed using the da Vinci Si or X platform (Intuitive Surgical, Inc., Sunnyvale, CA, USA), with an oncologic approach based on complete mesocolic excision (CME) and central vascular ligation [1]. Patients were placed supine, tilted slightly head-down and rotated toward the left side, and pneumoperitoneum was induced via Palmer’s point. Four 8-mm robotic trocars were arranged diagonally across the abdominal wall at a minimum spacing of 8 cm, complemented by a 12-mm assistant port placed in the left hemiabdomen. Once the mesenteric fold had been identified, the ileocolic vessels were isolated and divided at their origin using polymer locking clips (Weck^®^ Hem-o-lok^®^, Teleflex Incorporated, Wayne, PA, USA), followed by lymphadenectomy along the corresponding vascular pedicles. Adequate bowel perfusion was confirmed by indocyanine-green fluorescence angiography. The terminal ileum and transverse colon were then divided with a linear stapler, intracorporeal continuity was reestablished via a hand-sewn, side-to-side ileocolic anastomosis, and the specimen was retrieved through a suprapubic Pfannenstiel incision.

### Manuscript preparation

The study design, data interpretation, manuscript structure and conclusions were developed independently within the author team. Every author critically reviewed and approved the submitted version and accepts responsibility for the accuracy and integrity of the work reported herein.

## Results

### Demographic characteristics

The overall study cohort consisted of 229 patients, allocated to a training group (TG; *n* = 164) and a control group (CG; *n* = 65). No significant between-group difference emerged for sex (χ²(1) = 0.824, *p*=.364), BMI (U = 5146, z = 0.025, *p*=.980), American Society of Anesthesiologists score [34] (H(1) = 3.653, *p*=.056), vascular diseases (χ²(1) = 0.636, *p*=.425), and preoperative parameters (Table 1, Online resource 1, Table S1). Age differed significantly between groups (TG: 77 years [73.3 ± 13.3] vs. CG: 80 years [76.8 ± 12.3]; U = 6252, z = 2.041, *p*=.041; Cohen’s d = 0.272), prior abdominal surgery (TG: *n* = 90 [55.6%] vs. CG: *n* = 47 [72.3%]; χ²(1) = 5.44, *p*=.020; Phi = 0.155), and ECOG performance status (TG: *n* = 24 [15.5%] vs. CG: *n* = 4 [6.2%]; H(1) = 8.305, *p*=.004; Cohen’s d = 0.372; Table 1).

**Table 1:** Demographic Characteristics

	Valid cases	Total Cohort *n* = 229 Median {mean ± SD} or number (%)*	TG *n* = 164 Median {mean ± SD} or number (%)*	CG *n* = 65 Median {mean ± SD} or number (%)*	*p*-value^A^	d_Cohen_	Phi	Test statistic	df/Z
Age [years]	229	78 {74.6 ± 13}	77 {73.3 ± 13.3}	80 {76.8 ± 12.3}	0.041	0.272		6252	2.041
Gender [female]	229	123 (53.7)	85 (51.8)	38 (58.5)	0.364			0.824	1
BMI [kg m^− 2^]	223	25 {25.3 ± 6.3}	25 {25.1 ± 6.5}	25 {25.7 ± 5.8}	0.98			5146	0.025
ASA	221	3 {2.59 ± 0.55}	3 {2.63 ± 0.55}	2 {2.48 ± 0.53}	0.056			3.653	1
1		4 (1.7)	3 (1.9)	1 (1.5)					
2		87 (38)	55 (35.3)	32 (49.2)					
3		127 (55.5)	95 (60.9)	32 (49.2)					
4		3 (1.3)	3 (1.9)	0 (0)					
ECOG	220	1 {1.23 ± 1}	1 {1.35 ± 1}	1 {0.92 ± 0.92}	0.004	0.372		8.305	1
0		60 (26.2)	37 (23.9)	23 (35.4)					
1		85 (37.1)	55 (35.5)	30 (46.2)					
2		44 (19.2)	37 (23.9)	7 (10.8)					
3		28 (12.2)	24 (15.5)	4 (6.2)					
4		3 (1.3)	2 (1.3)	1 (1.5)					
Vascular Disease^a^	228	72 (31.4)	54 (33.1)	18 (27.7)	0.425			0.636	1
History of prior abdominal surgeries	227	137 (59.8)	90 (55.6)	47 (72.3)	0.02		0.155	5.44	1

*As appropriate. ^a^ including peripheral artery disease, coronary artery disease. ^A^ Statistics were realised by Fisher’s exact test, ANOVA or Mann-Whitney-*U*-Test, as appropriate

### Characteristics of the surgical procedures

There was no significant between-group difference in blood loss (U = 4807.5, z=-1.496, *p*=.135), intraoperative blood transfusions (χ²(1) = 2.898, *p*=.089), conversions (χ²(1) = 0.659, *p*=.417), additional minor or major resections (H(1) = 1.434, *p*=.231), minor and major intraoperative complications (χ²(1) = 0.011, *p*=.917; χ²(1) = 0.411, *p*=.522), ClassIntra classification (H(1) = 0.002, *p*=.967), restoration of bowel continuity (χ²(1) = 1.243, *p*=.265), stoma formation (χ²(2) = 1.569, *p*=.456), chemotherapy (χ²(1) = 0.254, *p*=.614), or choice of operating surgeon (H(1) = 3.6, *p*=.058). Operative time, however, was significantly longer in the TG than in the CG (TG: 218 min [229 ± 72] vs. CG: 183 min [183 ± 38]; U = 3096, z=-4.706, *p*<.001; Cohen’s d = 0.662; Table 2, Online resource 1, Table S2).

**Table 2:** Therapy Characteristics

	Valid cases	Total Cohort *n* = 229 Median {mean ± SD} or number (%)*	TG *n* = 164 Median {mean ± SD} or number (%)*	CG *n* = 65 Median {mean ± SD} or number (%)*	*p*-value^A^	d_Cohen_	Test statistic	df/Z
OR time [min]	224	204 {216 ± 67}	218 {229 ± 72}	183 {183 ± 38}	≤ 0.001	0.662	3096	-4.706
Blood Loss [ml]	224	50 {68 ± 118}	50 {73 ± 136}	50 {58 ± 58}	0.135		4807.5	-1.496
Blood transfusion intraoperative	227	7 (3.1)	7 (4.3)	0 (0)	0.089		2.898	1
Conversion	224	11 (4.8)	9 (5.7)	2 (3.1)	0.417		0.659	1
Additional Procedure	224	44 (19.2)	28 (17.6)	16 (24.6)	0.231		1.434	1
Minor^a^		20 (8.7)	12 (7.5)	8 (12.3)	0.257		1.286	1
Major^b^		24 (10.5)	16 (10)	8 (12.3)	0.622		0.243	1
Intraoperative Complication^c^	224							
Minor		37 (16.2)	26 (16.4)	11 (16.9)	0.917		0.011	1
Major		1 (0.4)	1 (0.6)	0 (0)	0.522		0.411	1
Establishing Continuity	224	221 (96.5)	156 (98.1)	65 (100)	0.265		1.243	1
Stoma formation	229	10 (4.3)	6 (3.6)	4 (6.2)	0.456		1.569	2
Chemotherapy	229	65 (28.4)	45 (27.4)	20 (30.8)	0.614		0.254	1

*As appropriate. ^a^ biopsy, atypical liver resection, intestinal suture. ^b^ total or partial resection of other organs. ^c^ according to ClassIntra-Classification. ^A^ Statistics were realised by Fisher’s exact test, Chi^2^ test, Man-Whitney U-Test or Kruskal-Wallis-test, as appropriate. n/a: not applicable

### Postoperative characteristics

There was no significant between-group difference in intensive care unit stay (U = 4352, z = 0.794, *p*=.427), minor and major complications (χ²(1) = 0.633, *p*=.426; χ²(1) = 0.558, *p*=.455), Clavien–Dindo complication score (H(1) = 0.033, *p*=.856), total blood transfusions (χ²(1) = 0.001, *p*=.979), 30-day mortality (χ²(1) = 0.404, *p*=.525), 6-month stoma-associated complications (H(1) = 2.409, *p*=.121), or hospitalizations due to stoma-related complications (U = 5024, z=-1.418, *p*=.156). Overall hospital stay, however, was significantly longer in the TG (TG: 12 days [15.9 ± 12.5] vs. CG: 9 days [12.5 ± 9.7]; U = 3669.5, z=-3.428, *p*<.001; Cohen’s d = 0.467; Table 3).

**Table 3:** Postoperative Characteristics

	Valid cases	Total Cohort *n* = 229 Median {mean ± SD} or number (%)*	TG *n* = 164 Median {mean ± SD} or number (%)*	CG *n* = 65 Median {mean ± SD} or number (%)*	*p*-value^A^	d_Cohen_	Test statistic	df/Z
Length of stay								
ICU [d]	208	0.0 {1.1 ± 4.4}	0 {1.2 ± 4.3}	0 {1 ± 4.7}	0.427		4352	0.794
In total [d]	226	11 {14.9 ± 11.8}	12 {15.9 ± 12.5}	9 {12.5 ± 9.7}	≤ 0.001	0.467	3669.5	-3.428
Any complication^a^	227							
Minor		58 (25.3)	44 (27)	14 (21.9)	0.426		0.633	1
Major		36 (15.7)	24 (14.7)	12 (18.8)	0.455		0.558	1
Clavien-Dindo-complication-score	227				0.856		0.033	1
I		11 (4.8)	9 (5.5)	2 (3.1)				
II		47 (20.5)	35 (21.5)	12 (18.8)				
IIIa		6 (2.6)	4 (2.5)	2 (3.1)				
IIIb		16 (7)	11 (6.7)	5 (7.8)				
IVa		5 (2.2)	4 (2.5)	1 (1.6)				
IVb		1 (0.4)	0 (0)	1 (1.6)				
V		8 (3.5)	5 (3.1)	3 (4.7)				
Blood transfusion in total	226	32 (14)	23 (14.2)	9 (14.1)	0.979		0.001	1
30-Day Mortality	227	6 (2.6)	5 (3.1)	1 (1.6)	0.525		0.404	1

^a^ according to Clavien-Dindo-Complication-Score. ^A^ Statistics were realised by Fisher’s exact test, Chi^2^ test, Man-Whitney U-Test or Kruskal-Wallis-test, as appropriate

### Surgical procedure related complications

As detailed in Table S3, surgical complications, anastomotic leakage, non-surgical complications and re-operation rates were all statistically comparable between groups. Specifically, surgical complications did not differ significantly between the TG and CG (TG: *n* = 41 [25.2%] vs. CG: *n* = 15 [23.4%]; χ²(1) = 0.073, *p*=.787). Anastomotic leakage grade likewise showed no significant between-group difference (H(1) = 1.109, *p*=.292). Non-surgical complications were comparable between both groups (TG: *n* = 45 [27.6%] vs. CG: *n* = 18 [28.1%]; χ²(1) = 0.006, *p*=.938). Re-operation rates were also comparable (TG: *n* = 17 [10.4%] vs. CG: *n* = 9 [11.5%]; χ²(1) = 0.598, *p*=.439).

### Histopathological characteristics

Tumor entity differed significantly between the two groups. The CG comprised 65 (100%) confirmed carcinomas, whereas the TG listed 153 (93.9%) carcinomas (H(1) = 4.151, *p*=.042; Cohen’s d=0.238). UICC classification, however, did not differ significantly between groups. The CG demonstrated a higher proportion of UICC stage II compared with the TG (TG: *n* = 61 (38.6%) vs. CG: *n* = 35 (53.8%)). The TG showed higher proportions of UICC 0 (TG: *n* = 4 (2.5%) vs. CG: *n* = 0 (0%)), UICC I (TG: *n* = 38 (24.1%) vs. CG: *n* = 12 (18.5%)), UICC III (TG: *n* = 41 (25.9%) vs. CG: *n* = 15 (23.1%)), and UICC IV (TG: *n* = 14 (8.9%) vs. CG: *n* = 3 (4.6%); H(1) = 0.001, *p*=.969). No significant between-group difference was found for the proportion of T4 tumors (TG: *n* = 41 (25.3%) vs. CG: *n* = 17 (26.2%); χ²(1) = 0.017, *p*=.895), number of resected lymph nodes (TG: *n* = 18.5 [20.6 ± 8.5] vs. CG: *n* = 18 [19.3 ± 6.3]; U = 5125, z=-0.454, *p*=.650), number of affected lymph nodes (TG: *n* = 0 [1.8 ± 5] vs. CG: *n* = 0 [1 ± 2.8]; U = 5047.5, z=-0.778, *p*=.437), R-status (TG: *n* = 160 (98.2%) vs. CG: *n* = 63 (98.4%); H(1) = 0.021, *p*=.886), or tumor size (TG: *n* = 45 [49 ± 28.9] vs. CG: *n* = 45 [53 ± 35.4]; U = 5332, z = 0.606, *p*=.545; Online resource 1, Table S4).

### CUSUM-analysis

**Fig. 2:**
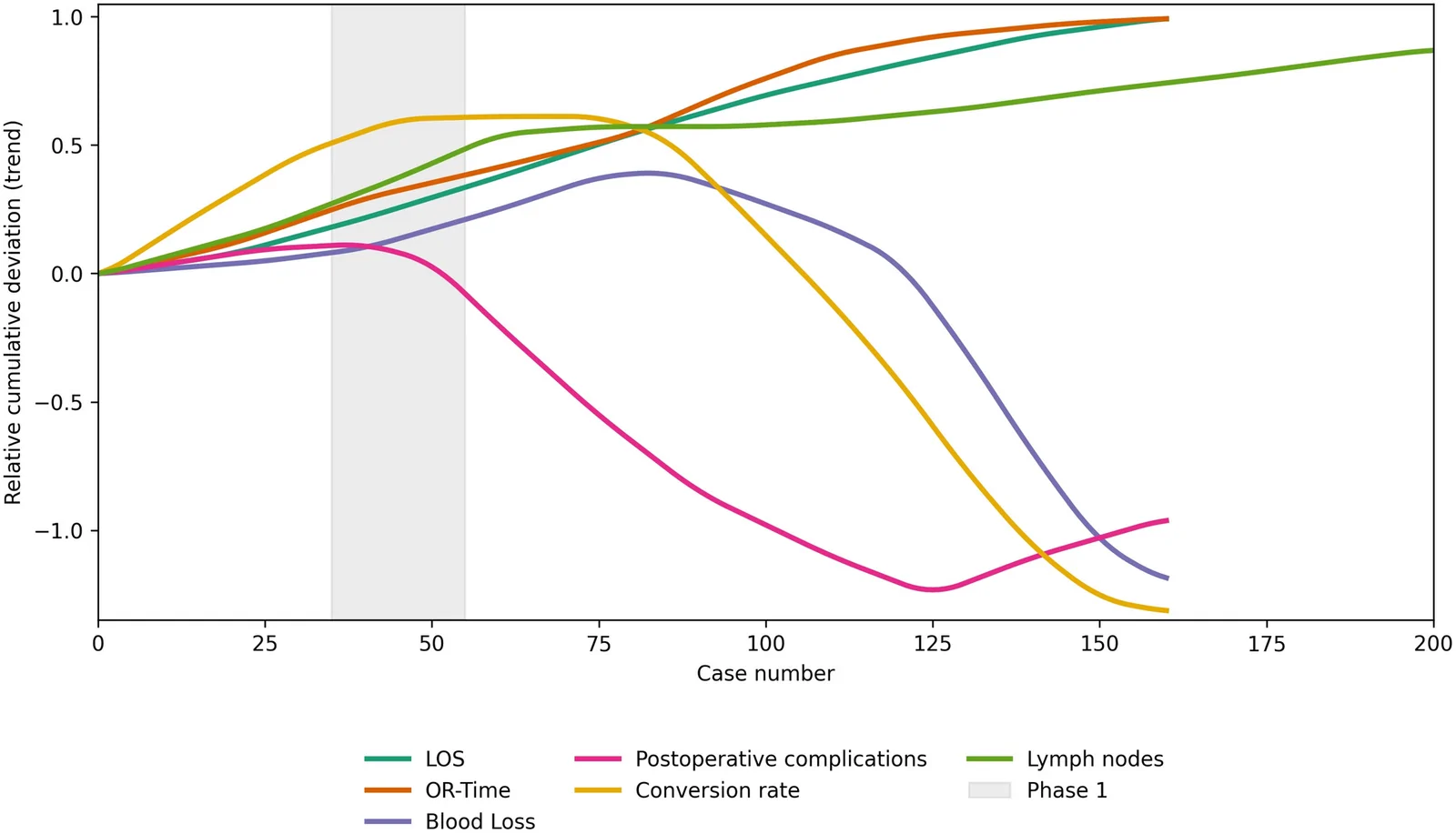
CUSUM analysis of the training group using external benchmark values derived from the control group for operative time, blood loss, lymph node yield, conversion rate, major postoperative complications, and length of stay. The initial learning phase was completed within an approximate transition zone of 35 to 55 procedures. Upward trends indicate performance below benchmark, whereas downward trends indicate performance exceeding benchmark values CUSUM = Cumulative sum analysis

The figure depicts CUSUM curves for LOS, OR-time, blood loss, lymph node yield, postoperative complications, and conversion rate, expressed as relative cumulative deviations across sequential case numbers. The shaded region identifies the phase 1 of the series and marks the approximate transition zone at case 35 to 55. The x-axis represents consecutive case numbers, while the y-axis denotes relative cumulative deviation. Most variables initially demonstrate an upward trajectory. LOS demonstrates sustained increase throughout the series, reaching its highest values toward the end of the observation period. OR-time shows a steady upward deviation across cases. Blood loss exhibits a modest initial increase above the reference line, reaching a peak at around 80 cases, followed by a progressive decline, crossing the zero line at around 125 cases and continuing into negative deviation toward the later cases. Lymph node yield shows a consistent upward trajectory from baseline that persists across the entire case sequence without major deviations. Postoperative complications demonstrate a slight initial rise above zero, followed by a pronounced downward deviation at around case 55 and subsequently showing a mild upward inflection (case 125), while remaining below the reference line. Conversion rate increases steadily in the early phase until reaching a plateau, after which it declines sharply (Fig. 2, Online resource 1, Figure S1).

### RA-CUSUM-analysis

**Fig. 3:**
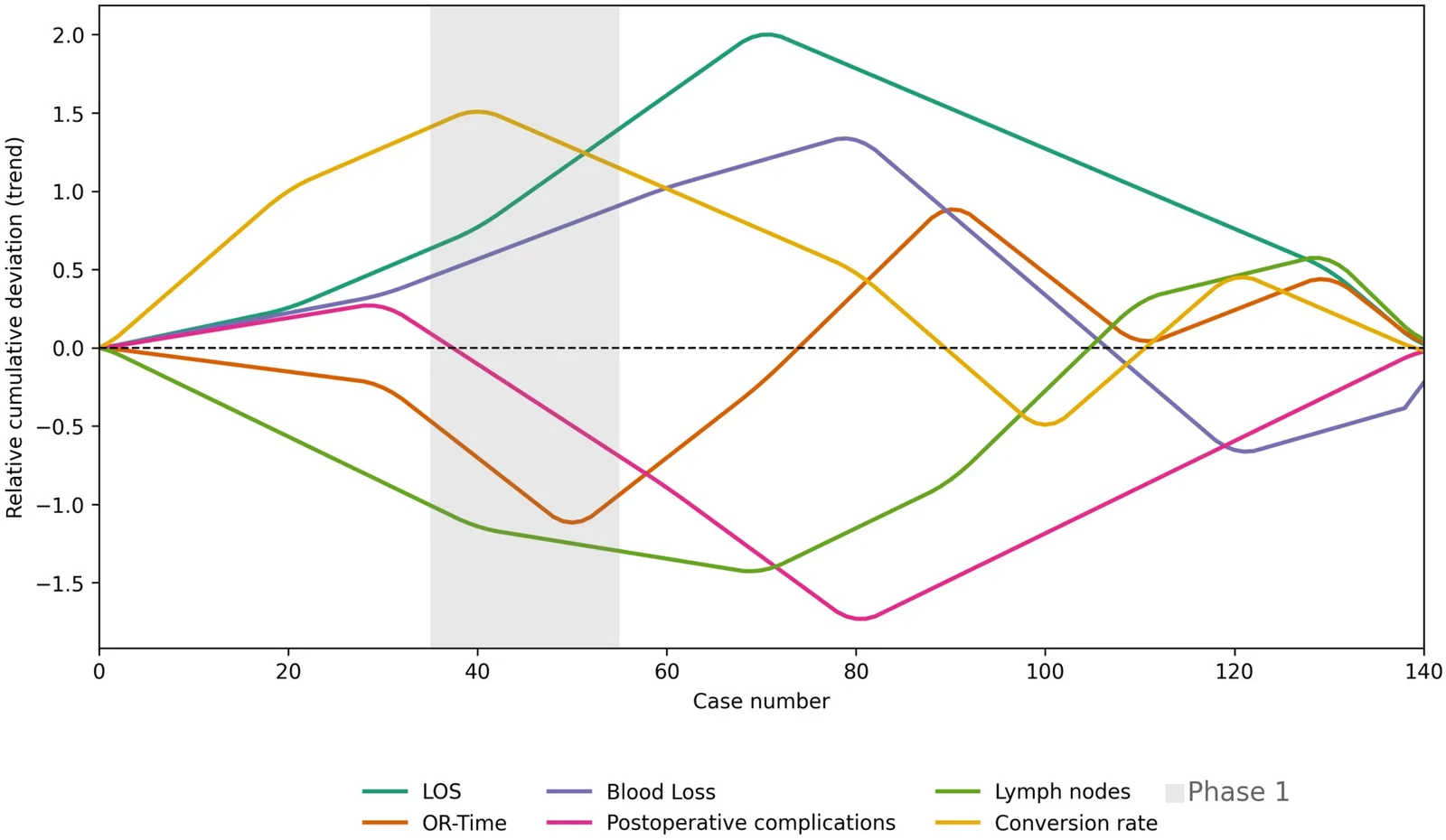
Risk-adjusted cumulative sum (RA-CUSUM) analysis of the training group adjusted for patient-related risk factors including age, BMI, ASA score, ECOG status, vascular comorbidities, prior abdominal surgery, and tumor size. RA-CUSUM curves were calculated for operative time, blood loss, conversion rate, major intraoperative complications, major postoperative complications, and length of stay. The prolonged first learning phase persisted after risk adjustment. Upward trends indicate performance below benchmark, whereas downward trends indicate performance exceeding benchmark values. RA-CUSUM = Risk-adjusted cumulative sum analysis, BMI = Body mass index, ASA = American Society of Anesthesiologists, ECOG = Eastern Cooperative Oncology Group

This graph presents RACUSUM curves for the variables LOS, OR-time, blood loss, lymph node yield, postoperative complications, and conversion rate. The shaded region identifies the phase 1 of the series and marks the approximate transition zone at case 35 to 55. The x-axis represents consecutive case numbers, while the y-axis denotes relative cumulative deviation (trend) with the horizontal dashed line indicating the reference. The trajectories appear more dispersed and less uniform around the zero reference line. LOS demonstrates an initial upward deviation from baseline, followed by a continued increase reaching a peak at around case 70 after which a gradual downward trajectory is observed. OR-time initially declines below the reference line. It rises subsequently at case 50, crosses the reference line at around case 70 and peaks at case 90 before declining again toward reference. Blood loss exhibits a steady upward trajectory reaching a maximum at case 80, followed by a decline that crosses the reference line at case 110 and continues into negative deviation before partially recovering toward the end. Lymph node yield initially decreases below the reference line, after which it demonstrates a progressive upward trend (case 70), crossing the reference line (around case 110) and continuing into positive deviation in the later cases. Postoperative complications show a gradual increase above the reference line in the early cases, followed by a sustained downward trajectory (case 35) reaching a pronounced negative deviation (case 80) and subsequently rising toward the reference line. Conversion rate demonstrates an early increase with a distinct peak at case 40 followed by a continuous decline, crossing the zero line and reaching negative deviation before returning toward the reference line in the final cases (Fig. 3, Online resource 1, Figure S2-S3).

Within the benchmark period, demographic and histopathological characteristics did not differ between the two consecutive years of the control group (Online resource 1, Table S5), indicating a stable case-mix across the time window from which the external benchmark was derived. Discrimination of the risk-adjustment model was moderate to good across the analysed outcomes (C-statistic 0.675–0.867), and calibration was adequate for all outcomes (Hosmer–Lemeshow *p* ≥ .105; Online resource 1, Table S6 and Figure S4).

Across the training-group case sequence, the proportion of T4 tumours increased (14.8% vs. 30.6%, *p* = .030) and patients were older (74 vs. 79 years, *p* = .004), whereas all further case-mix characteristics and operative time itself remained unchanged (Online resource 1, Table S7). A change point was present in all six CUSUM curves (Davies test, *p* ≤ .001 for all outcomes). For every outcome, a model with two change points was preferred over models with one or no change point. The first change point, marking the end of phase one, ranged from case 27 to case 80 with a median of case 42.5, and all change points exceeded the lower bound of previously reported transition ranges (Online resource 1, Table S8).

## Discussion

In this large single-center observational cohort study, we conducted a learning curve analysis using an external benchmark approach with increased emphasis on clinical correlation for robotic right-sided hemicolectomy. Application of this methodology revealed different learning curve patterns, particularly a longer first phase. In concrete terms, cohort-level analyses of the training group showed that major complications, conversion rate, blood loss, LOS, and lymph node yield exhibited the steepest rise in phase one, suggesting a transition between 35 and 55 cases (Figs. 2 and 3). However, this range should be interpreted as an approximate transition zone rather than a uniform cutoff across all outcomes, as some curves did not clearly stabilize within this interval. Objective change-point analysis supported this interpretation. A change point was demonstrable in every curve, and the median first change point at case 42.5 fell within the reported transition zone, which was thus centrally located within the distribution of outcome-specific change points (range 27 to 80). All change points exceeded the lower bound of the 20 to 35 cases reported previously. The position of the transition nevertheless varied between outcomes, which further supports the notion of an approximate transition zone rather than a fixed proficiency threshold. Because the training group was analysed at cohort level and case volumes differed substantially between surgeons, the reported transition zone describes aggregate learning behaviour and should be interpreted with caution when applied to an individual surgeon. After completing phase one complications decreased, while lymph node yield stabilized. Both parameters increased later, reflecting expanded indications and inclusion of additional surgeons. The curve for operative time ascended consistently, differing from the pattern of other variables. The continuous increase derives from the high median OR time in the control group, whose surgeons were already in the mastery phase. In addition, a progressive expansion of surgical indications towards more complex procedures over the course of the series may have contributed to this trend (Online resource 1, Figure S1-S3).

CUSUM analysis provides insight into procedural performance, but depends on investigators for interpretation [17, 19]. Based on a procedural definition different phases have been described, with the first phase ranging from 20 to 35 cases [2, 11, 20]. This differs from our findings and without linkage to clinical impact, such phase classifications remain technical and do not represent clinically meaningful learning curves. To the best of our knowledge, this is the first altered application of this methodology in surgical research. While later phases still reflect procedural classification, the first phase in RRH is longer than previously reported and marks the period during which perioperative and technical proficiency is attained [20]. We propose a reconsideration of learning curve analysis for RRH based on two principles. Firstly, the use of an external benchmark provides greater methodological consistency [18]. Secondly, process analyses should incorporate clinical outcomes to define patient-centered and therefore clinically relevant phases [35]. While applying these principles our findings suggest reconsideration of surgical training programs in RRH due to later attainment of perioperative and technical proficiency.

Despite the profound methodology and relevant findings, the results should be interpreted in light of several limitations. On the one hand the external benchmark derived from the two most experienced surgeons based on their most recent two years of practice. While this approach ensures the use of an external benchmark, the benchmark itself remains subjective and varies across cohorts. Due to differences in operative care, the benchmark defined at our institution is not generalizable. By reflecting the surgeons’ mastery phase, the benchmark represents a particularly high standard. Comprehensive analysis of RRH registry data may serve as a foundation for the development of a balanced and broader applicable benchmark.

Secondly, the boundary of the critical phase one was initially determined by the steepest increase in perioperative parameters in the CUSUM analysis and was subsequently corroborated by objective change-point analysis; the position of the transition nevertheless varied between outcomes. Random variation in their distribution across the learning curve phases cannot be excluded. Although the institutional database represents a substantial case volume by European standards, the sample size may still be insufficient to establish a robust correlation with clinical impact. Additionally, despite standardized data collection, analysis of a prospectively maintained database remains susceptible to incomplete documentation, information bias, as well as residual confounding. Owing to the limited number of events relative to the number of covariates, the risk-adjustment model should be regarded as explorative rather than predictive. Fourthly, the inclusion period introduced temporal effects independent of the surgical learning curve. Changes in perioperative management, the implementation of ERAS pathways and developments in oncologic treatment influenced outcomes over time. Enhanced recovery principles were first formalised for colonic surgery in 2005 [36] and were disseminated more broadly following the foundation of the ERAS Society in 2010, so that their establishment in routine practice falls within rather than before the inclusion period of the present study. Because the external benchmark was derived from the most recent two years of practice, it reflects contemporary perioperative care, whereas the earliest cases of the training group were performed under earlier standards. Part of the deviation observed during the initial phase may therefore reflect secular improvement in perioperative management rather than individual technical learning, and the length of the first phase reported here should be regarded as an upper estimate in this respect. Separating both influences would require a multi-institutional cohort in which surgeon experience and calendar time are not confounded. Finally, demographic characteristics differed slightly between the study cohorts; demographic characteristics showed a significantly higher ECOG in the study group whereas the control group was older and had more prior abdominal surgeries, counterbalancing this difference. Proficiency was inferred from perioperative parameters together with lymph node yield and resection margin status. These represent surrogates of oncologic quality; disease-free and overall survival were not incorporated into the learning-curve modelling, so the analysis cannot determine whether early-phase cases carry a higher long-term oncologic failure risk.

Taken together, these limitations should be considered; however, this study applies an external benchmark methodology with clinical focus, providing a basis for reevaluating learning curve analysis for RRH and a framework for future studies. We observed a prolonged initial phase, indicating delayed attainment of perioperative and technical proficiency. Consequently, learning curve analysis should shift to a clinically driven approach, requiring reevaluation of current paradigms and adaptation of surgical training programs.

## Conclusion

The prolonged first learning phase for RRH indicates delayed attainment of perioperative and technical proficiency and suggests that learning curve frameworks and surgical training programs should be reconsidered. External benchmark methodology with outcome emphasis enables clinically driven assessment of learning curves. This approach may serve as a conceptual framework for future analyses.

## Supplementary Information

Below is the link to the electronic supplementary material.

Supplementary Material 1

Supplementary Material 2

## Data Availability

Owing to patient-privacy and ethical constraints, the datasets generated and analyzed for this study are not deposited in a public repository; qualified researchers may request access from the corresponding author.
