# Supplementary material for: Limitations of procedural learning curves: reassessment of the clinical relevance of learning curve analysis in robotic right hemicolectomy
Source: J Robot Surg. 2026 Sep 24;20(1):1019. doi: 10.1007/s11701-026-03970-w (PMC13612698; doi:10.1007/s11701-026-03970-w)
Supplement: Supplementary file 1 [file 11701_2026_3970_MOESM1_ESM.pdf]

Supplementary Information

Title: Limitations of Procedural Learning Curves: Reassessment of the Clinical Relevance of Learning Curve Analysis in Robotic Right Hemicolectomy

Journal of Robotic Surgery

Alexander Wilk[1 2], Anna-Marie Wilk[3], Hatice Altin[4], Thorsten Brechmann[4 5], Metin Mazgaldzhi[4 6], Benno Mann[4 6]

Affiliations:

1 Faculty of Medicine, University of Tübingen, Tübingen, Germany

2 Department of General, Visceral and Transplant Surgery, University Hospital Tübingen, Tübingen, Germany

3 Wroclaw Medical University, Wroclaw, Poland

4 Ruhr University Bochum, Bochum, Germany

5 Department of Gastroenterology and Oncology, Knappschaft Kliniken Bottrop, Bottrop, Germany

6 Department of General, Visceral and Robotic Surgery, Augusta-Klinikum Bochum Mitte, Bochum, Germany

Address for correspondence:

Alexander Wilk, Department of General, Visceral and Transplant Surgery, University Hospital Tübingen, Hoppe-Seyler-Straße 3, 72076 Tübingen, Germany.
Email: alexander.wilk@med.uni-tuebingen.de. Tel.: +49 (0) 7071 29-86620, ORCID iD: 0009-0004-3127-0377.

Supplementary Materials - Index

Supplementary Tables

Table S1. Laboratory Parameters *pag. 3*

Table S2. Additional Therapy Characteristics *pag.4*

Table S3. Procedure related complications *pag. 5*

Table S4. Histopathological Characteristics *pag. 6*

Figure S1. CUSUM analysis, parameter overview *pag. 7*

Figure S2. RA-CUSUM analysis I, parameter overview *pag. 8*

Figure S3. RA-CUSUM analysis II, parameter overview *pag. 9*

Supplementary Tables

Table S1. Laboratory Parameters

	**Valid cases**	**Total Cohort n = 229** Median {mean ± SD} or number (%)*	**TG n = 164** Median {mean ± SD} or number (%)*	**CG n = 65** Median {mean ± SD} or number (%)*	***p*-value**[A]	**Test statistic**	**df/Z**
**Pre-Operative**							
Leukocytes (cells/ul)	133	7.6 {7.9 ± 2.7}	7.5 {7.8 ± 2.5}	8.5 {8.5 ± 3.2}	.17	1838.5	1.371
Hemoglobin [g/dL]	133	9.9 {10.2 ± 2.3}	9.7 {10.2 ± 2.2}	10.3 {10.3 ± 2.7}	.375	1747.5	.886
Thrombocytes [/ul]	133	283 {299 ± 114}	283 {299 ± 112}	283 {297 ± 120}	.938	1595.5	.077
Quick	134	92 {90 ± 16.2}	91 {91.1 ± 14.4}	93 {89 ± 21.2}	.702	1669	.383
Creatinine [mg/dl]	134	.9 {1 ± .33}	.9 {.99 ± .34}	1.1 {1 ± .31}	.06	1945	1.852
CRP (mg/L)	132	.65 {.2.4 ± .3.8}	.6 {2.1 ± 3.3}	.9 {3.2 ± 5.3}	.36	1698	.915
CEA (ng/mL)	71	2.8 {31 ± 147}	2.7 {31.5 ± 154}	7.3 {28.1 ± 58.7}	.375	270	.888
CA-19.9 (ng/mL)	70	11 {209 ± 1138}	11.6 {174 ± 1118}	8.1 {522 ± 1362}	.463	183	-.734
**Post- Operative**							
CEA [ng/mL]	8	1.6 {3 ± 4.3}	1.8 {3.4 ± 4.4}	n/a	.25	0	-1.537
CA-19.9 (ng/mL)	8	9.2 {11.1 ± 10.4}	10.6 {12.7 ± 10.1}	n/a	.25	0	-1.528

* As appropriate
[A] Statistics were realised by Fisher's exact test, ANOVA or Mann-Whitney-*U*-Test, as appropriate

Table S2. Additional Therapy Characteristics

	**Valid cases**	**Total Cohort n = 229** Median {mean ± SD} or number (%)*	**TG n = 164** Median {mean ± SD} or number (%)*	**CG n = 65** Median {mean ± SD} or number (%)*	***p*-value[A]**	**Test statistic**	**df/Z**
**ClassIntra Classification**	224				.967	.002	1
none		186 (81.2)	132 (83)	54 (83)			
Grad 1		33 (14.4)	23 (14.5)	10 (15.4)			
Grad 2		4 (1.7)	3 (1.9)	1 (1.5)			
Grad 3		1 (.4)	1 (.6)	0 (0)			
Grad 4		0 (0)	0 (0)	0 (0)			
Grad 5		0 (0)	0 (0)	0 (0)			
**Surgeon**	228				.058	3.6	1
1		117 (51.1)	92 (56.4)	25 (38.5)			
2		101 (44.1)	61 (37.4)	40 (61.4)			
3		3 (1.3)	3 (1.8)	0 (0)			
4		5 (2.2)	5 (3.1)	0 (0)			
Other		2 (.9)	2 (1.2)	0 (0)			

*As appropriate
[A] Statistics were realised by Fisher's exact test, Chi^2 test, Man-Whitney U-Test or Kruskal-Wallis-test, as appropriate
n/a: not applicable

Table S3. Procedure related complications

	Valid cases	Total Cohort n = 229 Median {mean ± SD} or number (%)*	TG n = 164 Median {mean ± SD} or number (%)*	CG n = 65 Median {mean ± SD} or number (%)*	*p*-value[A]	Test statistic	df/Z
**Surgical Complications[a]**	227	56 (24.5)	41 (25.2)	15 (23.4)	.787	.073	1
**Anastomotic leak[b]**	227				.292	1.109	1
Grad A		2 (.9)	1 (.6)	1 (1.6)			
Grad B		1 (.4)	1 (.6)	0 (0)			
Grad C		15 (6.6)	9 (5.5)	6 (9.4)			
**Non-surgical Complications[c]**	227	63 (27.5)	45 (27.6)	18 (28.1)	.938	.006	1
**Re-Operation**	227	26 (11.4)	17 (10.4)	9 (11.5)	.439	.598	1
**6-Months complications (stoma associated)**	227				.121	2.409	1
Renal failure		2 (.9)	2 (1.2)	0 (0)			
Re-operation		4 (1.7)	4 (2.5)	0 (0)			
**Hospitalization due to stoma complications [d]**	226	.0 {0 ± 0}	0 {.98 ± 6.9}	0 {0 ± 0}	.156	5024	-1.418

[a] Including bleeding, atony, anastomosis insufficiency, incisional hernia, wound infection, ileus, injury to other organs, ischemia, blood transfusion
[b] according to ISREC Classification
[c] Including DVT, PE, UTI, pneumonia, pleural effusion, electrolyte disturbances
[A] Statistics were realised by Fisher's exact test, Chi^2 test, Man-Whitney U-Test or Kruskal-Wallis-test, as appropriate

Table S4. Histopathological Characteristics

	**Valid cases**	**Total Cohort n = 229** Median {mean ± SD} or number (%)*	**TG n = 164** Median {mean ± SD} or number (%)*	**CG n = 65** Median {mean ± SD} or number (%)*	***p*-value**[A]	**d_{Cohen}**	**Test statistic**	**df/Z**
**Entity**	228				.042	.238	4.151	1
Carcinoma		218 (95.2)	153 (93.9)	65 (100)				
Adenoma		8 (3.5)	8 (4.9)	0 (0)				
Other		2 (.9)	2 (1.2)	0 (0)				
**UICC**	223				.969		.001	1
UICC 0		4 (1.7)	4 (2.5)	0 (0)				
UICC I		50 (21.8)	38 (24.1)	12 (18.5)				
UICC II		96 (41.9)	61 (38.6)	35 (53.8)				
UICC III		56 (24.5)	41 (25.9)	15 (23.1)				
UICC IV		17 (7.4)	14 (8.9)	3 (4.6)				
**T4**	227	58 (25.3)	41 (25.3)	17 (26.2)	.895		.017	1
**Lymph nodes**	229							
number removed		18 {20.6 ± 7.9}	18.5 {20.6 ± 8.5}	18 {19.3 ± 6.3}	.65		5125	-.454
number affected		0 {1.6 ± 4.5}	0 {1.8 ± 5}	0 {1 ± 2.8}	.437		5047.5	-.778
**R-Status**	227				.886		.021	1
R0		223 (97.4)	160 (98.2)	63 (98.4)				
R1		4 (1.7)	3 (1.8)	1 (1.6)				
**Tumorsize (mm)**	221	45 {50.9 ± 30.9}	45 {49 ± 28.9}	45 {53 ± 35.4}	.545		5332	.606

*As appropriate
[A] Statistics were realised by Fisher's exact test, Chi^2 test, Man-Whitney U-Test or Kruskal-Wallis-test, as appropriate

Figure S1. CUSUM analysis, parameter overview

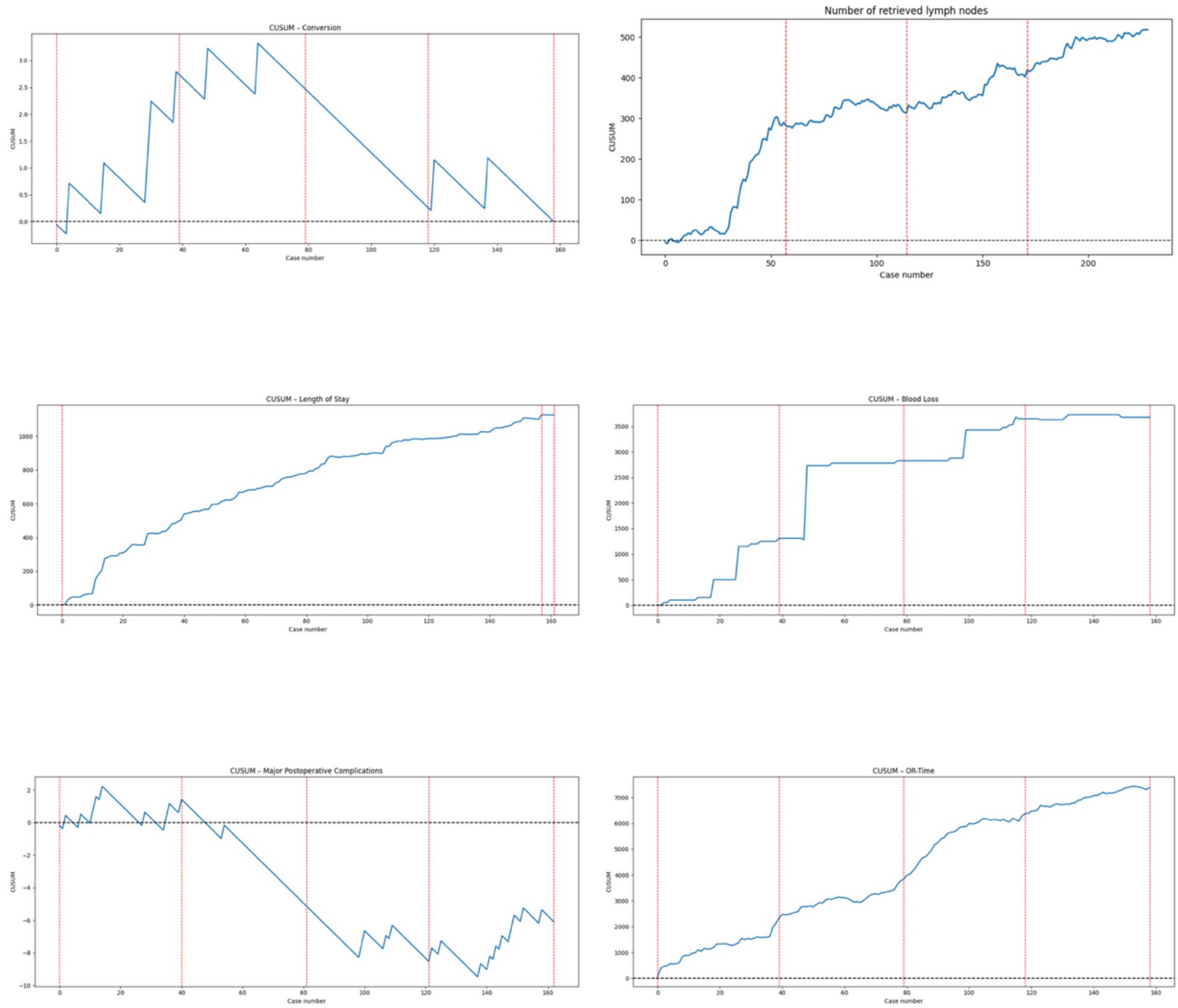

CUSUM analysis of the training group using external benchmark values derived from the control group for conversion rate, lymph node yield, length of stay, blood loss, major postoperative complications, and operative time. Red dashed lines indicate learning phase transitions.

CUSUM = Cumulative sum analysis

Figure S2. RA-CUSUM analysis I, parameter overview

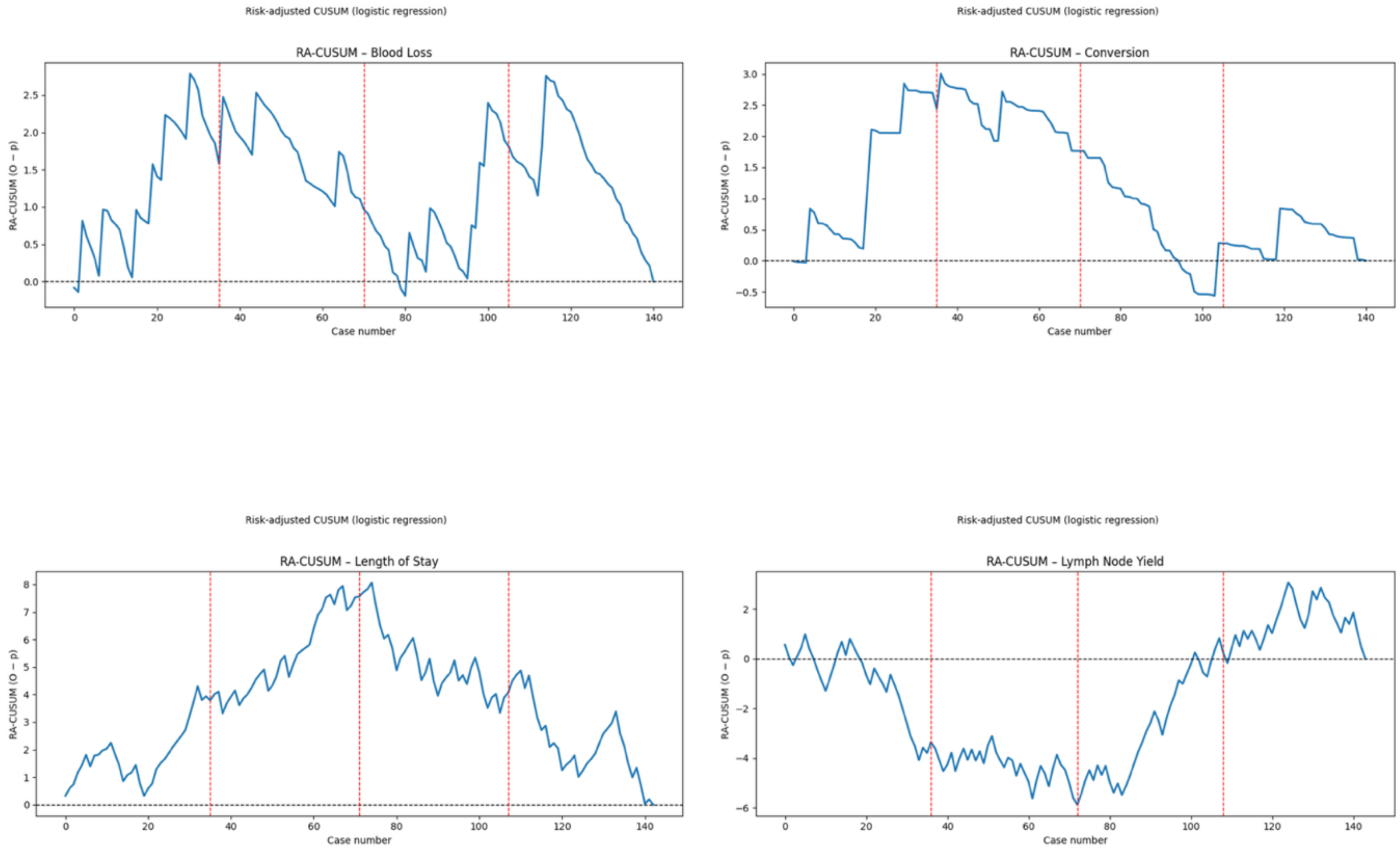

Risk-adjusted CUSUM analysis for blood loss, conversion rate, length of stay, and lymph node yield after adjustment for patient-related risk factors. Red dashed lines indicate learning phase transitions.

RA-CUSUM = Risk-adjusted cumulative sum analysis

Figure S3. RA-CUSUM analysis II, parameter overview

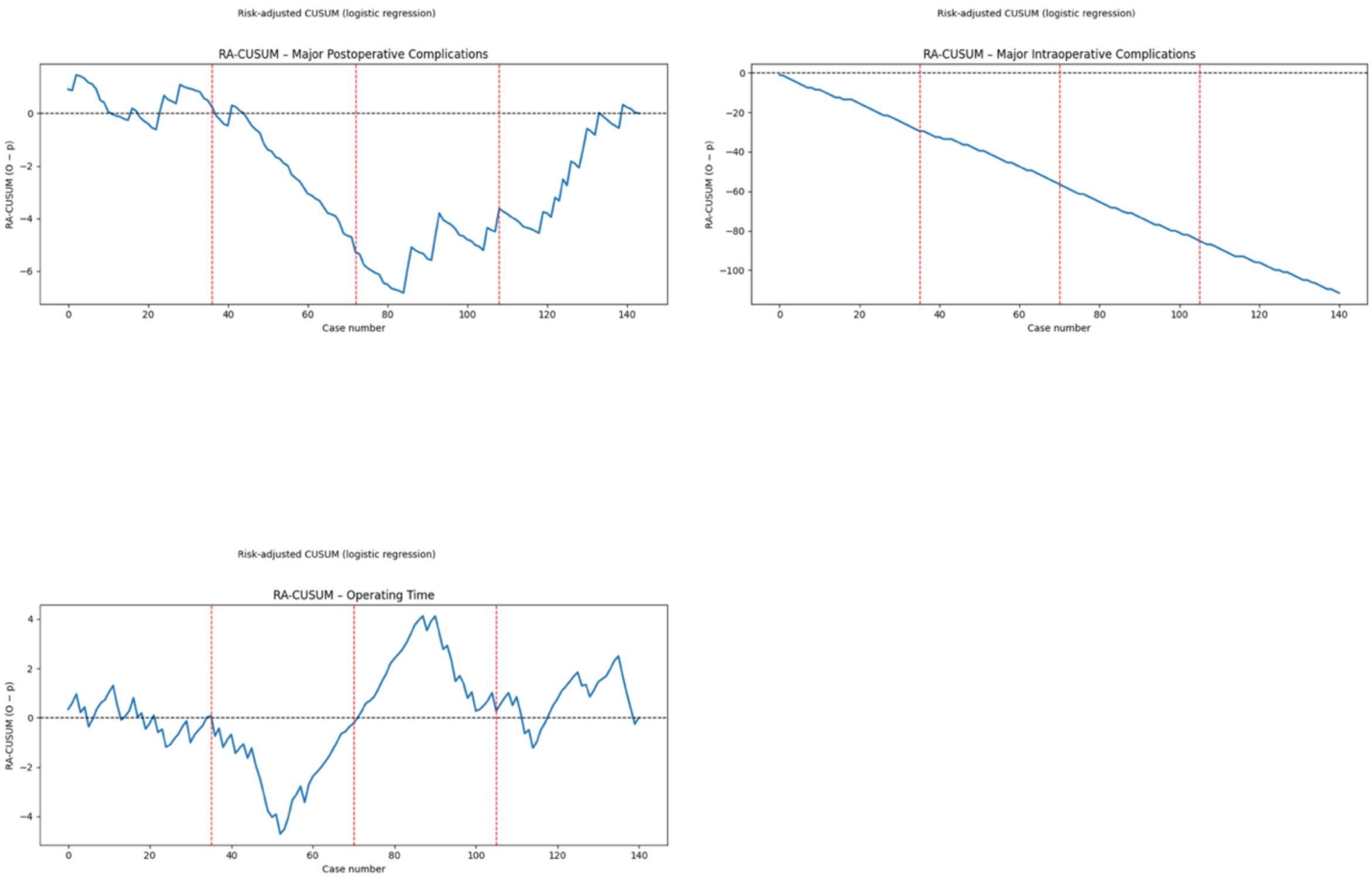

Risk-adjusted CUSUM analysis for major postoperative complications, major intraoperative complications, and operative time after adjustment for patient-related risk factors. Red dashed lines indicate learning phase transitions.

RA-CUSUM = Risk-adjusted cumulative sum analysis
