# Supplementary material for: Limitations of procedural learning curves: reassessment of the clinical relevance of learning curve analysis in robotic right hemicolectomy
Source: J Robot Surg. 2026 Sep 24;20(1):1019. doi: 10.1007/s11701-026-03970-w (PMC13612698; doi:10.1007/s11701-026-03970-w)
Supplement: Supplementary file 2 [file 11701_2026_3970_MOESM2_ESM.pdf]

Supplementary Information

Title: Limitations of Procedural Learning Curves: Reassessment of the Clinical Relevance of Learning Curve Analysis in Robotic Right Hemicolectomy

Journal of Robotic Surgery

Alexander Wilk[1 2], Anna-Marie Wilk[3], Hatice Altin[4], Thorsten Brechmann[4 5], Metin Mazgaldzhi[4 6], Benno Mann[4 6]

Affiliations:

1 Faculty of Medicine, University of Tübingen, Tübingen, Germany

2 Department of General, Visceral and Transplant Surgery, University Hospital Tübingen, Tübingen, Germany

3 Wroclaw Medical University, Wroclaw, Poland

4 Ruhr University Bochum, Bochum, Germany

5 Department of Gastroenterology and Oncology, Knappschaft Kliniken Bottrop, Bottrop, Germany

6 Department of General, Visceral and Robotic Surgery, Augusta-Klinikum Bochum Mitte, Bochum, Germany

Address for correspondence:

Alexander Wilk, Department of General, Visceral and Transplant Surgery, University Hospital Tübingen, Hoppe-Seyler-Straße 3, 72076 Tübingen, Germany. Email: alexander.wilk@med.uni-tuebingen.de. Tel.: +49 (0) 7071 29-86620, ORCID iD: 0009-0004-3127-0377.

Supplementary Materials – Index

Supplementary Tables	
Table S1. Laboratory Parameters	pag. 3
Table S2. Additional Therapy Characteristics	pag. 4
Table S3. Procedure related complications	pag. 5
Table S4. Histopathological Characteristics	pag. 6
Table S5. Baseline characteristics of the control group by year of surgery	pag. 7
Table S6. Performance of the risk-adjustment model	pag. 8
Table S7. Case-mix across the training-group case sequence	pag. 9
Table S8. Objective change-point analysis of the CUSUM curves	pag. 10
**Supplementary Figures**	
Figure S1. CUSUM analysis, parameter overview	pag. 11
Figure S2. RA-CUSUM analysis I, parameter overview	pag. 12
Figure S3. RA-CUSUM analysis II, parameter overview	pag. 13
Figure S4. Calibration of the risk-adjustment model	pag. 14

Supplementary Tables

Table S1. Laboratory Parameters

	**Valid cases**	Total Cohort<br>n = 229<br>Median {mean ± SD} or number (%)*	TG<br>n = 164<br>Median {mean ± SD} or number (%)*	CG<br>n = 65<br>Median {mean ± SD} or number (%)*	**p-value A**	**dCohen**	**Test statistic**	**df/Z**
Pre-Operative								
Leukocytes (cells/µl)	133	7.6 {7.9 ± 2.7}	7.5 {7.8 ± 2.5}	8.5 {8.5 ± 3.2}	.17		1838.5	1.371
Hemoglobin [g/dL]	133	9.9 {10.2 ± 2.3}	9.7 {10.2 ± 2.2}	10.3 {10.3 ± 2.7}	.375		1747.5	.886
Thrombocytes [/µl]	133	283 {299 ± 114}	283 {299 ± 112}	283 {297 ± 120}	.938		1595.5	.077
Quick	134	92 {90 ± 16.2}	91 {91.1 ± 14.4}	93 {89 ± 21.2}	.702		1669	.383
Creatinine [mg/dl]	134	.9 {1 ± .33}	.9 {.99 ± .34}	1.1 {1 ± .31}	.06		1945	1.852
CRP (mg/L)	132	.65 {2.4 ± 3.8}	.6 {2.1 ± 3.3}	.9 {3.2 ± 5.3}	.36		1698	.915
CEA (ng/mL)	71	2.8 {31 ± 147}	2.7 {31.5 ± 154}	7.3 {28.1 ± 58.7}	.375		270	.888
CA-19.9 (ng/mL)	70	11 {209 ± 1138}	11.6 {174 ± 1118}	8.1 {522 ± 1362}	.463		183	-.734
Post-Operative								
CEA [ng/mL]	8	1.6 {3 ± 4.3}	1.8 {3.4 ± 4.4}	n/a	.25		0	-1.537
CA-19.9 (ng/mL)	8	9.2 {11.1 ± 10.4}	10.6 {12.7 ± 10.1}	n/a	.25		0	-1.528

*As appropriate
A Statistics were realised by Fisher’s exact test, ANOVA or Mann-Whitney-U-Test, as appropriate
n/a: not applicable

Table S2. Additional Therapy Characteristics

	**Valid cases**	Total Cohort n = 229 Median {mean ± SD} or number (%)*	TG n = 164 Median {mean ± SD} or number (%)*	CG n = 65 Median {mean ± SD} or number (%)*	**p-value A**	**dCohen**	**Test statistic**	**df/Z**
ClassIntra Classification	224				.967		.002	1
none		186 (81.2)	132 (83)	54 (83)				
Grad 1		33 (14.4)	23 (14.5)	10 (15.4)				
Grad 2		4 (1.7)	3 (1.9)	1 (1.5)				
Grad 3		1 (.4)	1 (.6)	0 (0)				
Grad 4		0 (0)	0 (0)	0 (0)				
Grad 5		0 (0)	0 (0)	0 (0)				
Surgeon	228				.058		3.6	1
1		117 (51.1)	92 (56.4)	25 (38.5)				
2		101 (44.1)	61 (37.4)	40 (61.4)				
3		3 (1.3)	3 (1.8)	0 (0)				
4		5 (2.2)	5 (3.1)	0 (0)				
Other		2 (.9)	2 (1.2)	0 (0)				

*As appropriate
A Statistics were realised by Fisher's exact test, Chi2 test, Mann-Whitney U-Test or Kruskal-Wallis-test, as appropriate
n/a: not applicable

Table S3. Procedure related complications

	**Valid cases**	Total Cohort<br>n = 229<br>Median {mean ± SD} or number (%)*	TG<br>n = 164<br>Median {mean ± SD} or number (%)*	CG<br>n = 65<br>Median {mean ± SD} or number (%)*	**p-value A**	**dCohen**	**Test statistic**	**df/Z**
Surgical Complications a	227	56 (24.5)	41 (25.2)	15 (23.4)	.787		.073	1
Anastomotic leak b	227				.292		1.109	1
Grad A		2 (.9)	1 (.6)	1 (1.6)				
Grad B		1 (.4)	1 (.6)	0 (0)				
Grad C		15 (6.6)	9 (5.5)	6 (9.4)				
Non-surgical Complications c	227	63 (27.5)	45 (27.6)	18 (28.1)	.938		.006	1
Re-Operation	227	26 (11.4)	17 (10.4)	9 (11.5)	.439		.598	1
6-Months complications (stoma associated)	227				.121		2.409	1
Renal failure		2 (.9)	2 (1.2)	0 (0)				
Re-operation		4 (1.7)	4 (2.5)	0 (0)				
Hospitalization due to stoma complications [d]	226	.0 {0 ± 0}	0 {.98 ± 6.9}	0 {0 ± 0}	.156		5024	-1.418

*As appropriate
a Including bleeding, atony, anastomosis insufficiency, incisional hernia, wound infection, ileus, injury to other organs, ischemia, blood transfusion
b according to ISREC Classification
c Including DVT, PE, UTI, pneumonia, pleural effusion, electrolyte disturbances
A Statistics were realised by Fisher's exact test, Chi2 test, Mann-Whitney U-Test or Kruskal-Wallis-test, as appropriate

Table S4. Histopathological Characteristics

	**Valid cases**	Total Cohort n = 229 Median {mean ± SD} or number (%)*	TG n = 164 Median {mean ± SD} or number (%)*	CG n = 65 Median {mean ± SD} or number (%)*	**p-value A**	**dCohen**	**Test statistic**	**df/Z**
Entity	228				.042	.238	4.151	1
Carcinoma		218 (95.2)	153 (93.9)	65 (100)				
Adenoma		8 (3.5)	8 (4.9)	0 (0)				
Other		2 (.9)	2 (1.2)	0 (0)				
UICC	223				.969		.001	1
UICC 0		4 (1.7)	4 (2.5)	0 (0)				
UICC I		50 (21.8)	38 (24.1)	12 (18.5)				
UICC II		96 (41.9)	61 (38.6)	35 (53.8)				
UICC III		56 (24.5)	41 (25.9)	15 (23.1)				
UICC IV		17 (7.4)	14 (8.9)	3 (4.6)				
T4	227	58 (25.3)	41 (25.3)	17 (26.2)	.895		.017	1
Lymph nodes	229							
number removed		18 {20.6 ± 7.9}	18.5 {20.6 ± 8.5}	18 {19.3 ± 6.3}	.65		5125	-.454
number affected		0 {1.6 ± 4.5}	0 {1.8 ± 5}	0 {1 ± 2.8}	.437		5047.5	-.778
R-Status	227				.886		.021	1
R0		223 (97.4)	160 (98.2)	63 (98.4)				
R1		4 (1.7)	3 (1.8)	1 (1.6)				
Tumorsize (mm)	221	45 {50.9 ± 30.9}	45 {49 ± 28.9}	45 {53 ± 35.4}	.545		5332	.606

*As appropriate
A Statistics were realised by Fisher’s exact test, Chi2 test, Mann-Whitney U-Test or Kruskal-Wallis-test, as appropriate

Table S5. Baseline demographic and histopathological characteristics of the control group (external benchmark cohort) stratified by year of surgery

	**Valid cases**	CG total<br>n = 65<br>Median {mean ± SD} or number (%)*	CG 2024<br>n = 26<br>Median {mean ± SD} or number (%)*	CG 2025<br>n = 39<br>Median {mean ± SD} or number (%)*	**p-value A**	**Test statistic**	**df/Z**
Age [years]	65	80 {76.9 ± 12.4}	81 {76.7 ± 13.3}	78 {77.0 ± 11.9}	.893	517.5	.141
Gender [female]	65	38 (58.5)	14 (53.8)	24 (61.5)	.538	.38	1
BMI [kg m-2]	65	25 {25.7 ± 5.8}	26 {25.9 ± 5.3}	25 {25.6 ± 6.2}	.432	566	.792
ASA	65	2 {2.48 ± .53}	2 {2.38 ± .57}	3 {2.54 ± .51}	.300	1.073	1
1		1 (1.5)	1 (3.8)	0 (0.0)			
2		32 (49.2)	14 (53.8)	18 (46.2)			
3		32 (49.2)	11 (42.3)	21 (53.8)			
4		0 (0.0)	0 (0.0)	0 (0.0)			
ECOG	65	1 {.92 ± .92}	1 {.92 ± 1.02}	1 {.92 ± .87}	.817	.054	1
0		23 (35.4)	10 (38.5)	13 (33.3)			
1		30 (46.2)	11 (42.3)	19 (48.7)			
2		7 (10.8)	3 (11.5)	4 (10.3)			
3		4 (6.2)	1 (3.8)	3 (7.7)			
4		1 (1.5)	1 (3.8)	0 (0.0)			
Vascular Disease a	65	18 (27.7)	5 (19.2)	13 (33.3)	.213	1.549	1
History of prior abdominal surgeries	65	47 (72.3)	17 (65.4)	30 (76.9)	.308	1.037	1
Entity [carcinoma]	65	65 (100)	26 (100)	39 (100)	n/a	n/a	n/a
UICC	65	2 {2.14 ± .77}	2 {2.04 ± .77}	2 {2.21 ± .77}	.377	.782	1
I		12 (18.5)	6 (23.1)	6 (15.4)			
II		35 (53.8)	14 (53.8)	21 (53.8)			
III		15 (23.1)	5 (19.2)	10 (25.6)			
IV		3 (4.6)	1 (3.8)	2 (5.1)			
T4	65	17 (26.2)	6 (23.1)	11 (28.2)	.645	.212	1
Resected Lymphnodes	65	18 {19.3 ± 6.3}	20 {19.7 ± 7.1}	18 {19.1 ± 5.8}	.692	537	.403
Affected Lymphnodes	65	0 {1.1 ± 2.9}	0 {1.1 ± 2.9}	0 {1.1 ± 2.9}	.430	461	-.797
R-Status b	64	63 (98.4)	26 (100.0)	37 (97.4)	.404	.695	1
Tumorsize (mm)	65	45 {53.7 ± 35.4}	46 {53.3 ± 21.2}	45 {54.0 ± 42.7}	.361	575.5	.92

*As appropriate
a including peripheral artery disease, coronary artery disease
b R0 resection
A Statistics were realised by Chi2 test, Mann-Whitney U-Test or Kruskal-Wallis-test, as appropriate
CG = control group (external benchmark cohort); n/a: not applicable

Table S6. Performance of the logistic risk-adjustment model underlying the RA-CUSUM analysis, assessed in the training group (n = 164) for each analysed outcome a

	**Valid cases**	**Events n (%)**	**C-statistic (95 % CI) b**	**χ^2 c**	**df**	**p-value c**
OR time	141	102 (72.3)	.675 (.574–.776)	13.216	8	.105
Blood loss	141	16 (11.3)	.680 (.534–.810)	5.164	8	.740
Lymph node yield	144	66 (45.8)	.683 (.599–.770)	7.787	8	.455
Conversion	141	8 (5.7)	.867 (.755–.950)	3.196	8	.921
Major postoperative complications	144	21 (14.6)	.727 (.609–.836)	3.080	8	.929
Length of stay	143	99 (69.2)	.706 (.610–.794)	11.261	8	.187

a Adverse outcome defined relative to the external benchmark: OR time > 183 min, blood loss > 50 ml, lymph node yield < 18 nodes, length of stay > 9 days; for conversion and major postoperative complications the event itself
b Risk-adjustment model comprising age, BMI, ASA, ECOG, vascular disease, prior abdominal surgery and tumour size; 95 % CI obtained by stratified bootstrap resampling (2000 replicates)
c Hosmer–Lemeshow goodness-of-fit test
CI = confidence interval

Table S7. Case-mix characteristics and operative time across the chronological case sequence of the training group

	**Valid cases**	Cases 1–55<br>n = 55<br>Median {mean ± SD} or number (%)*	Cases 56–164<br>n = 109<br>Median {mean ± SD} or number (%)*	**p-value A**	**Test statistic**	**df/Z**	**ρ B**	**p-value B**
Age [years]	164	74 {71.7 ± 10.1}	79 {74.7 ± 14.7}	.004	2160	-2.919	.140	.073
BMI [kg m-2]	158	26 {26.8 ± 6.4}	25 {24.4 ± 6.5}	.176	3031	1.357	-.099	.217
ASA	156	3 {2.55 ± .58}	3 {2.66 ± .55}	.235	1.408	1	.008	.925
ECOG	155	1 {1.39 ± 1.16}	1 {1.33 ± 1.00}	.864	.029	1	-.037	.648
History of prior abdominal surgeries	162	28 (52.8)	62 (56.9)	.626	.237	1	.014	.859
T4	162	8 (14.8)	33 (30.6)	.030	4.719	1	.152	.054
UICC	158	2 {2.00 ± 1.00}	2 {2.21 ± .95}	.309	1.035	1	.078	.329
Tumorsize (mm)	156	45 {48.4 ± 26.4}	46 {50.6 ± 30.3}	.730	2637	-.347	.066	.414
Additional procedure	159	11 (20.8)	17 (16.0)	.462	.542	1	-.017	.829
Major additional resection	159	6 (11.3)	10 (9.4)	.709	.139	1	.029	.720
OR time [min]	159	220 {237.4 ± 86.9}	218 {225.8 ± 65.0}	.864	2801	.173	-.099	.215

*As appropriate
A Statistics were realised by Chi2 test, Mann-Whitney U-Test or Kruskal-Wallis-test, as appropriate
B Spearman rank correlation between the chronological case number within the training group and the respective variable, across all 164 cases
Cases were ordered chronologically by date of surgery; the cut-off at case 55 corresponds to the upper limit of the transition zone identified in the CUSUM analysis

Table S8. Objective change-point analysis of the CUSUM curves in the training group

	Valid cases	Davies statistic a	p-value a	BIC, no change point	BIC, one change point	BIC, two change points	First change point (95 % CI) b	Second change point b	Model selected c
OR time	159	8.684	≤.001	-958.2	-1015.8	-1230.8	80 (78–82)	95	two
Blood loss	159	21.196	≤.001	-646.9	-857.4	-910.7	40 (35–44)	55	two
Lymph node yield	164	24.353	≤.001	-644.8	-892.9	-1059.3	29 (27–31)	50	two
Conversion	159	27.087	≤.001	-514.5	-786.2	-837.0	65 (62–67)	110	two
Major postoperative complications	163	14.446	≤.001	-570.7	-701.7	-826.2	45 (41–49)	99	two
Length of stay	162	29.119	≤.001	-857.7	-1151.7	-1345.2	27 (25–29)	90	two

a Davies test for the presence of a change point (null hypothesis: no change point)
b Change points estimated by segmented (piecewise linear) regression using exhaustive least-squares search; 95 % confidence intervals for the first change point obtained by residual bootstrap (1000 replicates), conditional on the second change point
c Model selection between zero, one and two change points by the Bayesian information criterion
Case numbers refer to the chronological case sequence of the training group. Across the six outcomes, the first change point had a median of case 42.5 (range 27–80)
BIC = Bayesian information criterion; CI = confidence interval

Supplementary Figures

Figure S1. CUSUM analysis, parameter overview

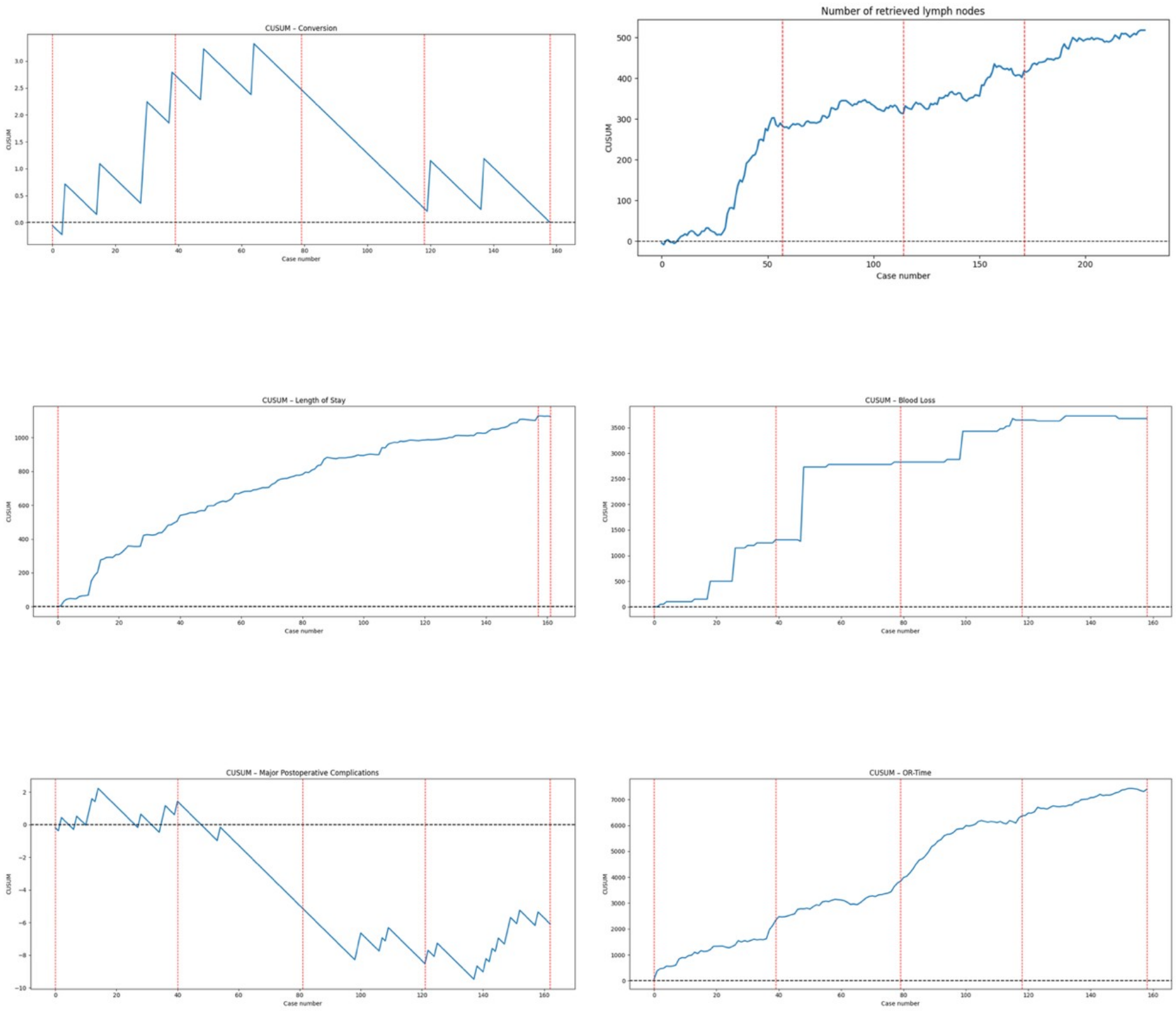

CUSUM analysis of the training group using external benchmark values derived from the control group for conversion rate, lymph node yield, length of stay, blood loss, major postoperative complications, and operative time. Red dashed lines indicate learning phase transitions.

CUSUM = Cumulative sum analysis

Figure S2. RA-CUSUM analysis I, parameter overview

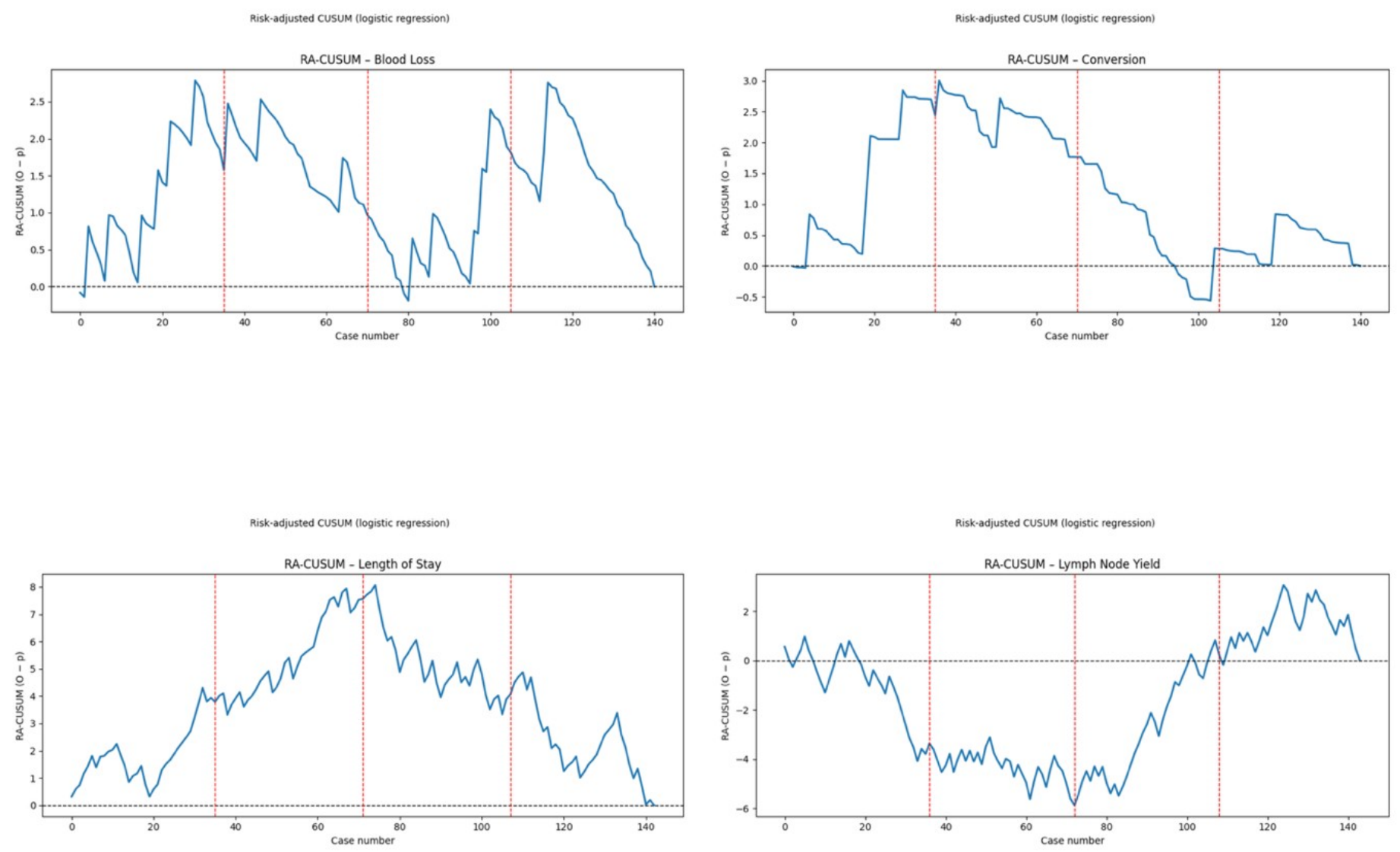

Risk-adjusted CUSUM analysis for blood loss, conversion rate, length of stay, and lymph node yield after adjustment for patient-related risk factors. Red dashed lines indicate learning phase transitions.

RA-CUSUM = Risk-adjusted cumulative sum analysis

Figure S3. RA-CUSUM analysis II, parameter overview

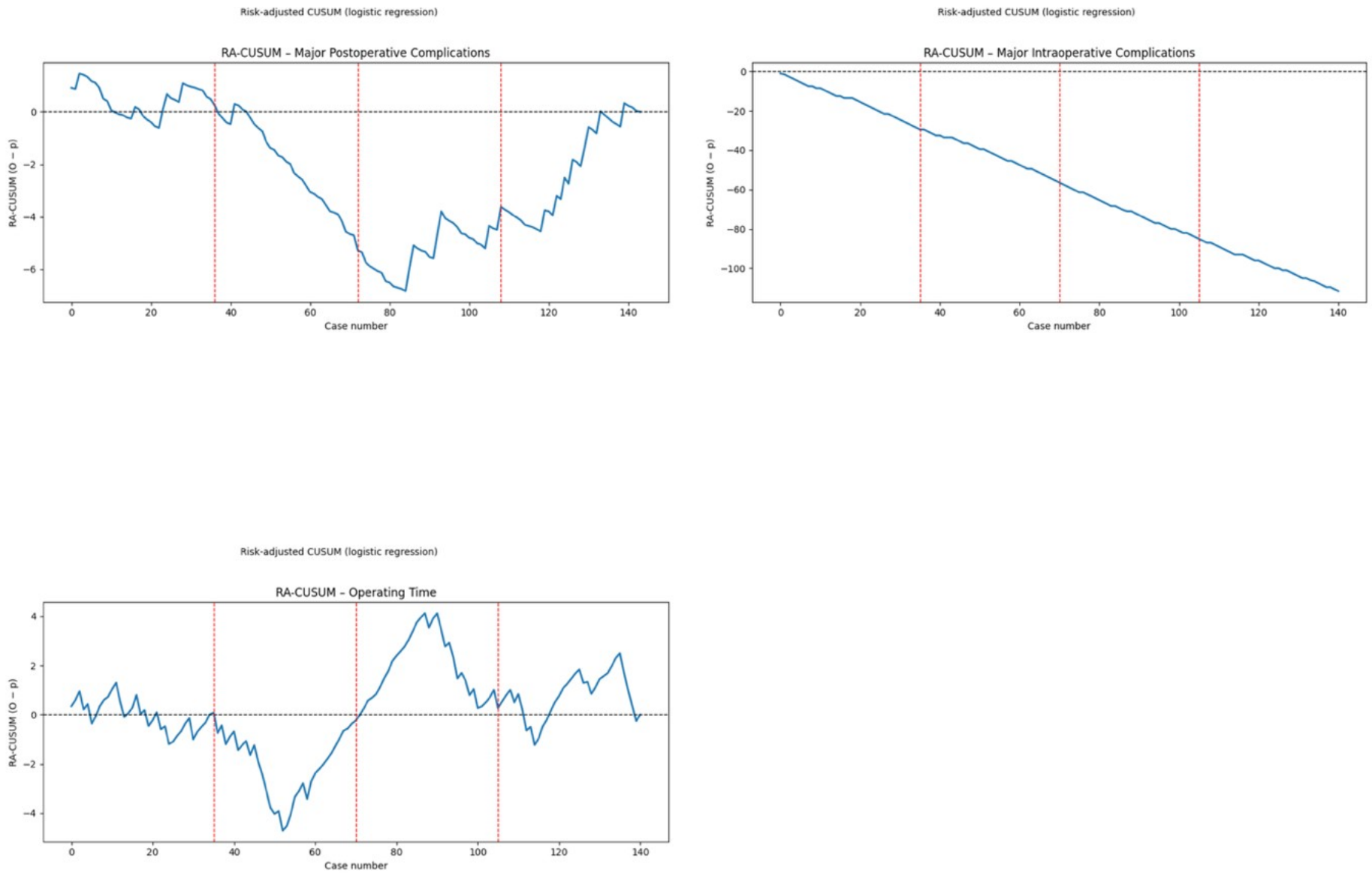

Risk-adjusted CUSUM analysis for major postoperative complications, major intraoperative complications, and operative time after adjustment for patient-related risk factors. Red dashed lines indicate learning phase transitions.

RA-CUSUM = Risk-adjusted cumulative sum analysis

Figure S4. Calibration of the logistic risk-adjustment model underlying the RA-CUSUM analysis, shown for all six analysed outcomes in the training group

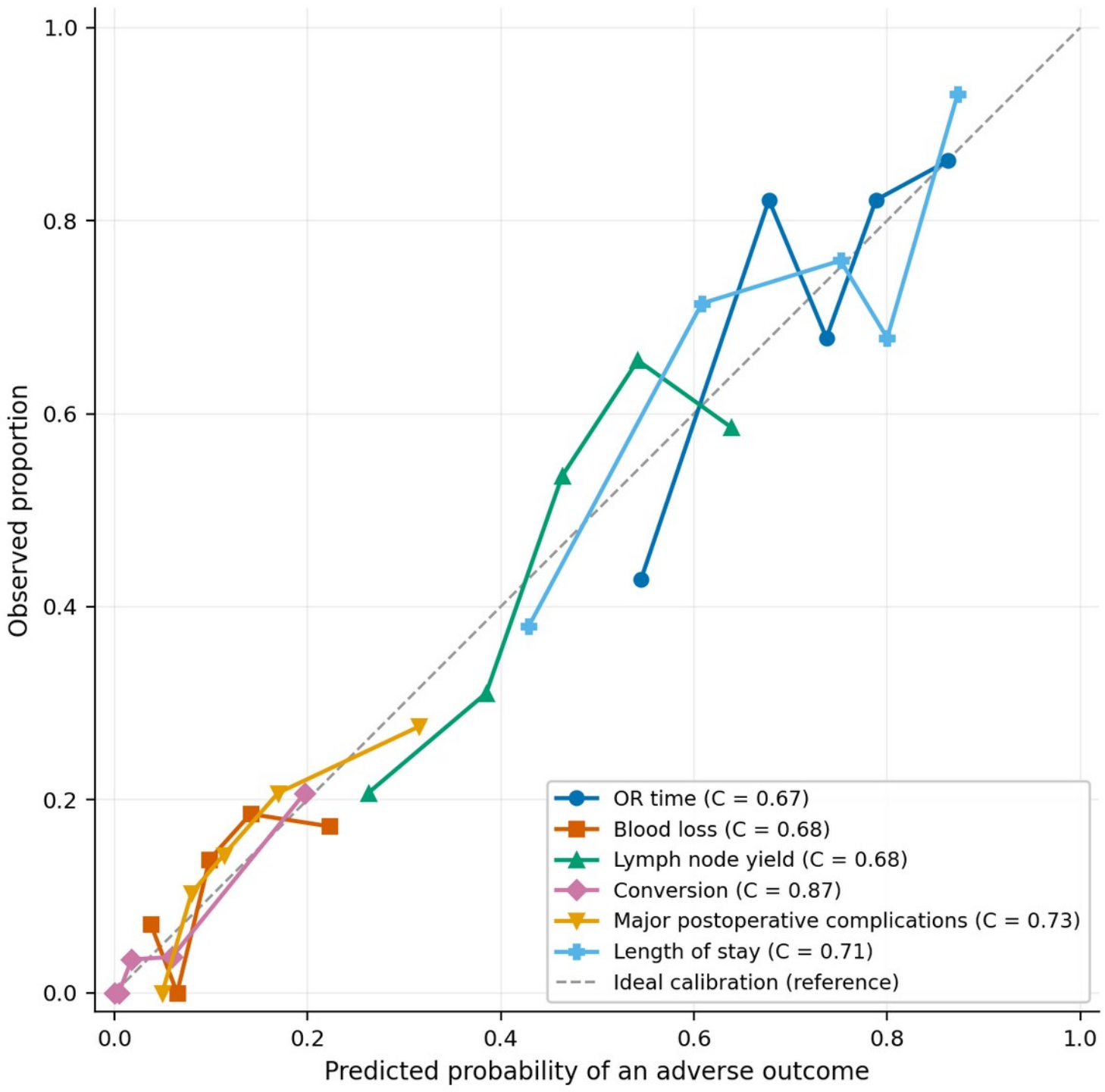

Observed proportions are plotted against predicted probabilities in quintiles of predicted risk. The dashed line denotes ideal calibration, on which observed and predicted rates coincide. C = C-statistic (area under the receiver operating characteristic curve). Adverse outcomes were defined relative to the external benchmark as detailed in Table S6.

RA-CUSUM = Risk-adjusted cumulative sum analysis; CI = confidence interval
